# Evaluation of the indications for performing magnetic resonance imaging of the female pelvis at a referral center for cancer, according to the American College of Radiology criteria

**DOI:** 10.1590/0100-3984.2015.0123

**Published:** 2017

**Authors:** Camila Silva Boaventura, Daniel Padilha Rodrigues, Olimpio Antonio Cornehl Silva, Fabrício Henrique Beltrani, Rayssa Araruna Bezerra de Melo, Almir Galvão Vieira Bitencourt, Gustavo Gomes Mendes, Rubens Chojniak

**Affiliations:** 1 MD, Imaging Department, A.C.Camargo Cancer Center, São Paulo, SP, Brazil.; 2 MD, Resident in the Imaging Department, A.C.Camargo Cancer Center, São Paulo, SP, Brazil.; 3 PhD, MD, Imaging Department, A.C.Camargo Cancer Center, São Paulo, SP, Brazil.; 4 PhD, MD, Head of the Imaging Department, A.C.Camargo Cancer Center, São Paulo, SP, Brazil.

**Keywords:** Magnetic resonance imaging, Female pelvis, Oncology

## Abstract

**Objective:**

To evaluate the indications for performing magnetic resonance imaging of the female pelvis at a referral center for cancer.

**Materials and Methods:**

This was a retrospective, single-center study, conducted by reviewing medical records and imaging reports. We included 1060 female patients who underwent magnetic resonance imaging of the pelvis at a cancer center between January 2013 and June 2014. The indications for performing the examination were classified according to the American College of Radiology (ACR) criteria.

**Results:**

The mean age of the patients was 52.6 ± 14.8 years, and 49.8% were perimenopausal or postmenopausal. The majority (63.9%) had a history of cancer, which was gynecologic in 29.5% and nongynecologic in 34.4%. Of the patients evaluated, 44.0% had clinical complaints, the most common being pelvic pain (in 11.5%) and bleeding (in 9.8%), and 34.7% of patients had previously had abnormal findings on ultrasound. Most (76.7%) of the patients met the criteria for undergoing magnetic resonance imaging, according to the ACR guidelines. The main indications were evaluation of tumor recurrence after surgical resection (in 25.9%); detection and staging of gynecologic neoplasms (in 23.3%); and evaluation of pelvic pain or of a mass (in 17.1%).

**Conclusion:**

In the majority of the cases evaluated, magnetic resonance imaging was clearly indicated according to the ACR criteria. The main indication was local recurrence after surgical treatment of pelvic malignancies, which is consistent with the routine protocols at cancer centers.

## Abstract (pt)

**Objetivo:**

Comparar as indicações de ressonância magnética da pelve feminina num centro de referência oncológico.

**Materiais e Métodos:**

Estudo retrospectivo, unicêntrico, realizado por revisão de prontuários e laudos de exames de imagem. Foram incluídas 1060 pacientes que realizaram ressonância magnética da pelve, de janeiro de 2013 a junho de 2014, num centro oncológico. As indicações dos exames foram classificadas segundo os critérios do American College of Radiology (ACR).

**Resultados:**

A idade média das pacientes foi 52,6 ± 14,8 anos, com 49,8% na perimenopausa ou pós-menopausa. A maioria (63,9%) apresentava antecedente de câncer, sendo 29,5% ginecológicos e 34,4% não ginecológicos. Das pacientes com queixa clínica (44%), os sintomas mais relatados foram dor pélvica (11,5%) e sangramento (9,8%). Em 34,7% das pacientes havia alteração ultrassonográfica prévia. A maioria das pacientes (76,7%) apresentou indicação adequada para o exame, segundo os critérios do ACR. As principais indicações foram: avaliação da recorrência tumoral após ressecção (25,9%), detecção e estadiamento de neoplasias ginecológicas (23,3%) e avaliação de dor ou massa pélvica (17,1%).

**Conclusão:**

A maioria dos exames avaliados apresentou indicação adequada segundo os critérios do ACR. A principal indicação foi a pesquisa de recidiva local após tratamento cirúrgico de neoplasias pélvicas, compatível com a rotina num centro oncológico.

## INTRODUCTION

Magnetic resonance imaging (MRI) is an increasingly popular imaging method in medical practice. It allows the acquisition of multiplanar images, with high resolution, without exposure to radiation, and offers the option of using a paramagnetic contrast agent (gadolinium). In oncology, MRI can provide morphological information such as size, contours, number of lesions, edema, necrosis, relationship to adjacent structures, physiological alterations, and cellular metabolism, allowing a more complete evaluation in terms of disease distribution and activity. MRI has been increasingly indicated for the management of cancer patients in Brazil^(1-11)^.

MRI applied to gynecology offers additional information on the anatomy of the female pelvis in comparison with other imaging modalities, such as ultrasound and computed tomography (CT). Therefore, MRI is the method of choice for the diagnosis and staging, as well as for the evaluation of the treatment response and the detection of relapse after treatment, of gynecologic neoplasms. Non-oncologic indications for MRI of the female pelvis include inconclusive pelvic ultrasound examination, evaluation of postoperative complications, pelvic pain, malformations of the vagina or uterus, and pelvic floor defect^(12-17)^. The American College of Radiology (ACR) compiled these indications into an educational guide and guideline for clinical decision-making in medical practice^(18)^.

To our knowledge, there have been no nationwide surveys on the main indications for MRI of the female pelvis in Brazil. Therefore, it is essential to describe the experience of a national referral center for cancer in the use of pelvic MRI for the management of patients with gynecologic tumors in order to design future research projects, develop more coherent flowcharts, and devise study protocols that are more focused on specific indications.

The objective of this study was to evaluate the indications for MRI of the female pelvis at a referral center for cancer, in comparison with the criteria proposed by the ACR.

## MATERIALS AND METHODS

This was a retrospective, single-center, descriptive study, carried out through a review of medical charts and imaging reports. The study was approved by the research ethics committee of the institution. The imaging department of the institution conducts approximately 1400 examinations per month. Of those, approximately 10% are MRI studies of the female pelvis. We evaluated female patients who underwent MRI of the pelvis between January 2013 and June 2014 at a referral center for cancer. Cases in which the physician order did not list an indication for the examination were excluded, as were those for which there was no electronic medical record available at the institution.

For all of the patients included, an electronic questionnaire was completed. The questionnaire was designed to collect data regarding demographic characteristics (age and gender), the MRI protocol (intravenous or intravaginal administration of contrast medium), and clinical status, as well as a detailed description of the indication for the examination. In patients who had a confirmed diagnosis or were under high clinical suspicion of malignant neoplasm, the examinations were classified as being indicated for oncological purposes (oncologic indication). The examinations in patients who had no such previous diagnosis and presented low clinical suspicion for malignancy, the examinations were classified as being indicated for non-oncological purposes (non-oncologic indication). In cases with an oncologic indication, the primary tumor site and the reason for the examination (diagnosis, staging, response evaluation, or posttreatment follow-up) were evaluated.

The indications for the examination were further divided, according to the ACR criteria^(18)^, into the following groups: detection and staging of gynecologic neoplasms; evaluation of pelvic pain or a pelvic mass; identification of congenital anomalies; determination of the number, location, and type of fibroids; detection of pelvic floor defects; detection and staging of other nongynecologic pelvic tumors; assessment of the recurrence of pelvic tumors; evaluation of postoperative complications; determination of arterial or venous anatomy and patency; identification and staging of soft tissue sarcomas; identification of the source of lower abdominal pain in pregnant women; assessment of fetal or placental abnormalities; identification of inflammatory bowel disease and its complications; and planning of guidance for minimally invasive surgery and brachytherapy.

The information collected via the electronic questionnaire was exported to a Microsoft Excel-based database. Data were processed with the SPSS Statistics software package, version 20.0 (IBM Corp.; Armonk, NY, USA). We calculated descriptive statistics, adopting the usual measures of central tendency and dispersion for numerical variables, as well as absolute and relative frequencies for categorical variables.

## RESULTS

A total of 1060 MRI scans of the female pelvis were included during the study period. The mean age of the patients was 52.6 ± 14.8 years (range, 8-90 years). Of the 1060 patients, 693 (65.4%) were perimenopausal or postmenopausal. A history of cancer was noted in 678 patients (63.9%). The cancer was gynecologic in 313 patients (29.5%) and nongynecologic in 365 (34.4%).

The most common specialties of the requesting physicians were gynecology and obstetrics (*n* = 418; 39.4%); clinical oncology (*n* = 313; 29.5%); urology (*n* = 58; 5.5%); abdominal surgery (*n* = 57; 5.4%); and pelvic surgery (*n* = 18; 1.7%). The remaining 196 MRIs (18.6%) were requested by physicians from other specialties.

Of the 1060 patients evaluated, 594 (56.0%) were asymptomatic at the time of the MRI. Therefore, 466 patients (44.0%) had clinical complaints. The most commonly reported symptoms were pelvic pain, in 122 (11.5%), and bleeding, in 104 (9.8%). Pelvic ultrasound prior to MRI was reported in 425 patients (40.1%), and the ultrasound had revealed alterations in 368 (34.7%), the most common finding being adnexal mass, which had been observed in 153 (40.8%). Intravenous contrast was used in almost all of the examinations (92.8%), whereas intravaginal contrast was used in only 4.4%.

**Figure 1:**
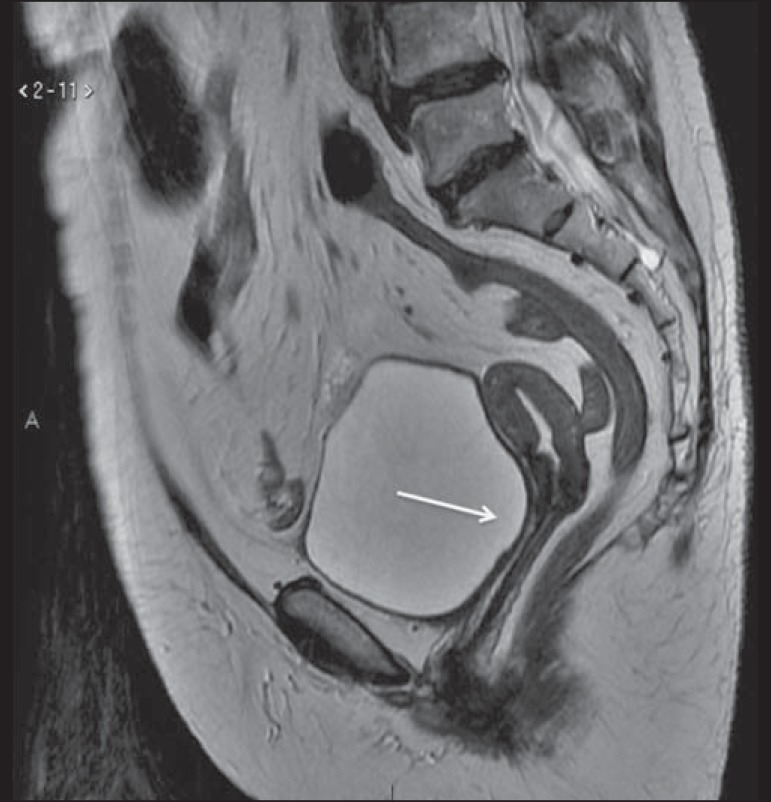
MRI scan requested for the assessment of pelvic tumor recurrence in a patient with cervical cancer after cone biopsy. T2-weighted sagittal section showing the uterus in anteversion, midline, with signs of surgical manipulation of the colon, and a diffuse reduction of T2 signal intensity.

**Figure 2:**
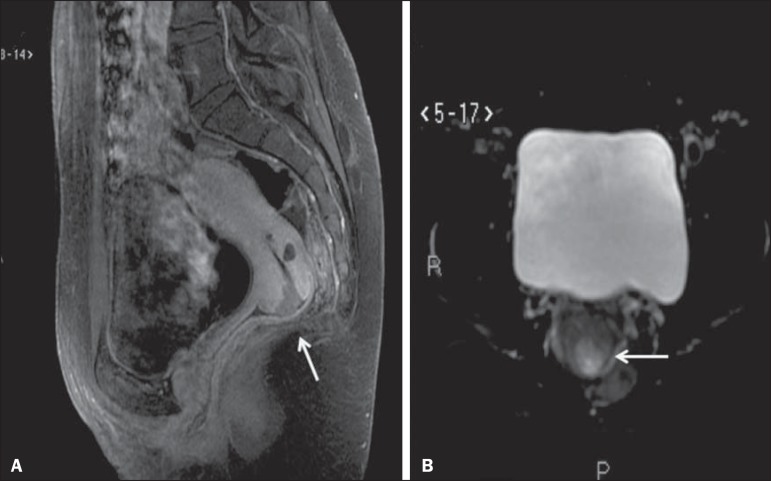
MRI scan requested for the detection/staging of a gynecologic neoplasm (cervical mass). **A:** Post-contrast T1-weighted sagittal section showing an irregular infiltrative lesion restricted to the cervix. **B:** Axial diffusion-weighted image showing diffusion restriction.

**Figure 3:**
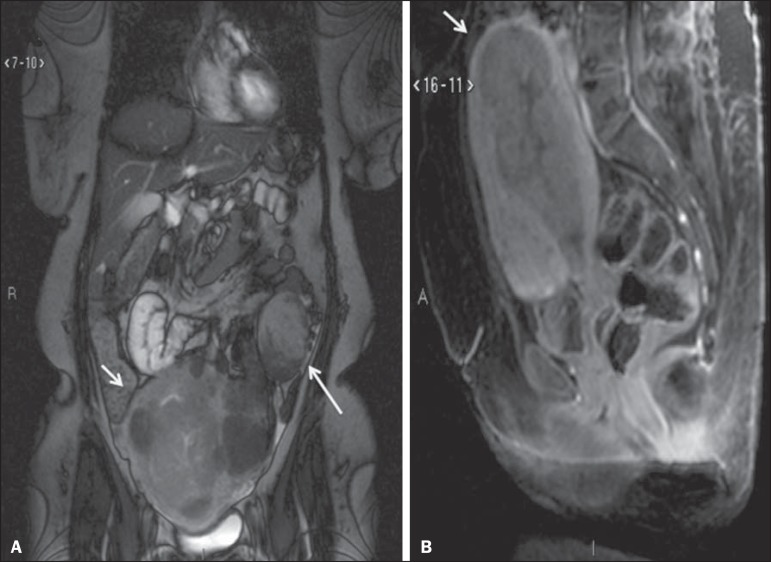
MRI scan requested for the diagnosis of an adnexal mass. Contrast-enhanced coronal T2-weighted slice (**A**) and sagittal T1-weighted slice (**B**), showing a heterogeneous infiltrative lesion occupying the endometrial cavity, with invasion to > 50% of the myometrial thickness (thick arrow) and solid-cystic mass in the left ovary (thin arrow), consistent with endometrial adenocarcinoma with metastasis to an ovary.

Of the 1060 patients evaluated, 813 (76.7%) presented an appropriate indication for MRI, according to the ACR criteria. The main indications observed were evaluation of tumor recurrence after resection, in 275 (25.9%), as shown in Figure 1; detection and staging of gynecologic neoplasms, in 247 (23.3%), as shown in Figure 2; and evaluation of pelvic pain or a pelvic mass, in 181 (17.1%), as shown in Figure 3. Table 1 describes the frequency of all indications according to the ACR criteria.

**Table 1:** Frequency of indications for MRI of the female pelvis according to the criteria established the ACR (*n* = 1060).

ACR criterion	N	%
Evaluation of recurrence of pelvic tumors	275	25.9
Detection and staging of gynecologic neoplasms	247	23.3
Assessment of pelvic pain or a pelvic mass	181	17.1
Detection and staging of other malignant tumors of the pelvis	59	5.6
Evaluation of fibroids	19	1.8
Identification and staging of soft tissue sarcomas	17	1.6
Evaluation of complications after pelvic surgery	7	0.7
Identification of congenital anomalies	3	0.3
Determination of arterial or venous anatomy and patency	2	0.2
Assessment of pelvic floor defects	1	0.1
Evaluation of abdominal pain in pregnant women	1	0.1
Identification of inflammatory bowel disease and its complications	1	0.1
Other	247	3.3

The indications for pelvic MRI examinations were categorized as oncologic in 763 patients (72.0%) and as nononcologic in 297 (28.0%). Of the non-oncologic indications, the most common were adnexal masses, in 89 patients (29.2%), leiomyomas, in 70 (23.0%), and adenomyosis or endometriosis, in 46 (15.1%). Among the oncologic indications, the most common primary tumors were ovarian cancer, in 145 patients (19.7%), cervical cancer, in 110 (15.0%), and endometrial cancer, in 74 (10.1%). Also among the oncologic indications, the examination was performed for follow-up/evaluation of post-treatment relapse, in 421 patients (55.2%); for diagnosis, in 176 (23.1%); for staging, in 94 (12.3%); for evaluation of the treatment response, in 46 (6.0%); or for other purposes, in 26 (3.4%). Table 2 describes the indications for MRI in patients with the most common primary tumors evaluated in our sample.

**Table 2:** Frequency of the MRI examination of the female pelvis in the patients with the most common types of gynecologic cancer (*n* = 324).

	Diagnosis or staging			Response evaluation			Follow-up			Total	
Type of cancer	N	%		N	%		N	%		N	%
Cervical	35	32.4		4	3.7		69	63.9		108	100
Endometrial	37	51.3		4	5.6		31	43.1		72	100
Ovarian	75	52.1		3	2.1		66	45.8		144	100

## DISCUSSION

The results of the present study demonstrate that, for the majority of the pelvic MRI scans evaluated in the present study, the indication for the examination was appropriate according to the ACR criteria. In our sample of female patients treated at a referral center for cancer, the main indication for MRI was the evaluation of post-treatment tumor recurrence, followed by the diagnosis and staging of gynecologic tumors.

MRI is widely accepted as a reliable imaging technique for the diagnosis, staging, and post-treatment follow-up of patients with gynecologic neoplasms. It plays a strategic role in the initial assessment, as well as in treatment planning, and is more effective than are other imaging techniques, especially in evaluating the invasion of adjacent structures and lymph node involvement^(19-23)^.

In addition to the morphological evaluation, with highcontrast resolution for evaluation of soft tissues, MRI makes it possible to obtain functional information that is associated with histological and prognostic factors. The functional evaluation is already part of the standard protocol for MRI of the female pelvis, the most widely used methods being dynamic post-contrast evaluation and diffusion-weighted imaging. Those methods contribute to more accurate characterization of the lesions, staging (especially in the evaluation of small peritoneal implants and extrauterine masses), evaluation of treatment responses, and differentiation between post-treatment changes and tumor recurrence^(24,25)^.

Although the evaluation of post-treatment tumor recurrence was the most common indication in the present study, it is worth noting that there is no evidence to support the use of MRI or other routine imaging techniques for posttreatment follow-up. In general, imaging examinations should be reserved for patients with alterations on the physical examination or with elevated or symptomatic levels of the tumor marker CA-125^(26)^. However, such examinations are routinely requested in clinical practice. In a survey of United Kingdom oncology gynecologists, 43% of the respondents reported using MRI in cervical cancer patients, whereas 14% reported using the technique in ovarian cancer patients^(27)^. MRI is also frequently requested for the evaluation of recurrence of other nongynecologic pelvic tumors, especially in patients with colorectal cancer^(28)^.

In our sample, the most common non-oncologic indication was the evaluation of an adnexal mass. Although ultrasound is the method of choice in such cases, MRI can provide additional information when the ultrasound findings are inconclusive. MRI allows better characterization of the soft tissues of adnexal masses, with an accuracy of 91-93% for differentiating between benign and malignant lesions^(29-31)^.

Other common non-oncologic indications include the assessment of leiomyomas or adenomyosis/endometriosis. MRI is the most accurate imaging technique for the identification and evaluation of leiomyomas, providing information about the location, vascularization, and degeneration, thus facilitating the treatment planning^(32)^. In the study of adenomyosis, MRI has a reported diagnostic accuracy of 85%^(33,34)^. For patients with suspected endometriosis, MRI helps assess the extent of disease, especially in the detection of deep implants, which are difficult to characterize by other methods^(35,36)^.

The results of this study have a direct impact on clinical practice. Knowledge of the main indications for pelvic MRI examination allows the radiologist to follow appropriate examination protocols for each patient and to develop the skills needed in order to provide the necessary information for the proper management of each case. For example, according to the findings of this study, it is fundamental that the radiologist who analyzes this type of examination has knowledge of the current staging, as well as the recurrence patterns of the gynecologic tumors. However, these findings should be considered in the context of certain limitations. This was a retrospective study, based on the review of medical records and physician requests, the main limitations of which are due to incomplete data recording. In addition, because our results depict the routine at a referral center for cancer, they cannot be generalized to centers that serve other populations.

In conclusion, the majority of the MRI examinations evaluated presented an appropriate indication according to the ACR criteria. The main indications were the investigation of local recurrence after surgical treatment of pelvic neoplasms and the diagnosis/staging of gynecologic tumors, which is consistent with the routine at a cancer center. Future studies may evaluate the relationship between the indication for the test and its results for each specific disease, contributing to a better rationalization of the use of MRI in Brazil.
